# Magnetization Dynamics of Amorphous Ribbons and Wires Studied by Inductance Spectroscopy

**DOI:** 10.3390/ma4010037

**Published:** 2010-12-23

**Authors:** Israel Betancourt

**Affiliations:** Departamento de Materiales Metálicos y Cerámicos, Instituto de Investigaciones en Materiales, Universidad Nacional Autónoma de México, D.F. 04510, Mexico; E-Mail: israelb@correo.unam.mx; Tel.: +52-55-56224641; Fax: +52-55-56161371

**Keywords:** amorphous alloys, magnetization dynamics, magnteoinductance, amorphous ribbons, amorphous wires

## Abstract

Inductance spectroscopy is a particular formulation variant of the well known complex impedance formalism typically used for the electric characterization of dielectric, ferroelectric, and piezoelectric materials. It has been successfully exploited as a versatile tool for characterization of the magnetization dynamics in amorphous ribbons and wires by means of simple experiments involving coils for sample holding and impedance analyzer equipment. This technique affords the resolution of the magnetization processes in soft magnetic materials, in terms of reversible deformation of pinned domain walls, domain wall displacements and spin rotation, for which characteristic parameters such as the alloy initial permeability and the relaxation frequencies, indicating the dispersion of each process, can be defined. Additionally, these parameters can be correlated with chemical composition variation, size effects and induced anisotropies, leading to a more physical insight for the understanding of the frequency dependent magnetic response of amorphous alloys, which is of prime interest for the development of novel applications in the field of telecommunication and sensing technologies. In this work, a brief overview, together with recent progress on the magnetization dynamics of amorphous ribbons, wires, microwires and biphase wires, is presented and discussed for the intermediate frequency interval between 10 Hz and 13 MHz.

## 1. Introduction

Amorphous alloys obtained from rapid solidification techniques as thin ribbons (with characteristic thickness below 30 microns and variable lengths) and fine wires (with typical diameters lower than 120 microns and variable lengths) have been the subject of research since the 1980s due to their ultrasoft magnetic character which has allowed interesting technological applications in devices for conversion of electromagnetic energy into mechanic energy and for signal processing; power electronics, electric power conditioning, magnetic sensors, telecommunication, automotive magnetics; and electronic article surveillance [1,2]. Amorphous ribbons can be cast by means of chill block melt spinning method which involves the continuous ejection of a molten alloy onto the edge surface of a wide rotating copper disk (with typical angular speeds within the range 2,000–3,000 rpm) which allows solidification rates between 10^5^–10^7^ K/s [3]. On the other hand, for the formation of wires with diameters within the range 80–120 microns, a variant of melt spinning process known as “rotating water bath melt spinning” entails the streaming of a fine jet of molten alloy into a flow of cold water (moving in a rapidly rotating drum at almost the same velocity as the jet) such that the alloy rapidly solidifies and vitrifies [4]. In addition, amorphous glass coated microwires, with usual diameters from 5 to 40 microns for the metallic nucleus and between 5–25 microns for the surrounding glass layer, can be produced by a modified Taylor-Ulitovsky technique in which a metal drop melted by high frequency induction is placed inside a glass tube. The temperature achieved by the melting assembly is high enough to allow the softening of the glass tube and its further drawing to a thin capillary which in turn is wound onto a receiving bobbin. The fast moving capillary entrapping the melted alloy passes through a cooling water jet for its rapid solidification [5].

Usually, within the study of soft magnetic materials, the characterization of the magnetic permeability *μ* as a function of frequency is a figure of merit because of the possibility for developing high frequency applications for telecommunications, wireless systems and radar detection, among others. In this context, a powerful characterization technique is the so called *impedance spectroscopy* (ImS) which has been widely used to investigate electric polarization phenomena in a variety of dielectric, ferroelectric, and piezoelectric materials [6]. However, it can be shown that by a simple change in experimental conditions (essentially coils instead of electrodes for applying external ac magnetic fields *h*_ac_ of variable intensity), and by a convenient transformation of complex formalisms (inductances and magnetic permeabilities instead of capacitances and permittivities), it is possible to build a methodology which can be established as *inductance spectroscopy* (InS) [7]. As in ImS, for materials characterization by InS, it is feasible to resolve the dominant magnetization processes by measuring in a wide frequency range. At low frequencies, all the magnetization processes (namely reversible domain wall bulging, domain wall displacement, spin rotation and spin precession) contribute to the total value permeability (and thus, of magnetization). However, they possess different dynamics in the sense that each magnetization process is characterized by a time-constant. As the frequency of *h*_ac_ increases, the magnetization processes with slow dynamics (*i.e*., large time-constants) become unable to follow the excitation field exhibiting a dispersion and subsequently, a decrease in the total value of permeability. The permeability value in each frequency range, the frequency at which the process ceases to follow the field, and the dispersion (the behavior to change from one magnetization process to another one) are significant aspects to understand the frequency response of the material. A wide variety of magnetic materials have been investigated by InS, such as ferrites [8], amorphous and nanocrystallized ribbons [9], amorphous wires [10] and glass-coated microwires [11].

The InS formalism involves the calculation of the complex inductance **L** = *L*_re_ + i*L*_im_ ( i = √-1) from complex impedance measurements **Z** = *Z*_re_ + iZ_im_ via the following transformation

**L** = −i**Z**/ω (1)

where ω is the angular frequency of *h_ac_*. Complementarily, the complex permeability formalism **μ** = μ_re_ + iμ_im_ can be formulated according to

**μ** = *G***L** (2)

where *G* is an appropriate geometrical factor. For instance, G~10^8^ (Henry)^−1^ for wires and microwires [12]. Within both of these formalisms, the real component *L*_re_ (or *μ*_re_) corresponds to the material magnetic permeability, whilst *L*_im_ (or *μ*_im_) is associated with the dissipative process. The spectroscopic plots *L*_re_ (ω) and *L*_im_ (ω) (or alternatively *μ*_re_ (ω) and *μ*_im_ (ω)) obtained from Equations (1) and (2) have led to the resolution as a function of frequency of the following magnetization mechanisms: Reversible domain wall bulging, unpinning-displacement of domain walls (*i.e*., hysteresis) and spin rotation. At frequencies low enough, all these magnetization processes contribute to the material’s response; in particular, domain wall movements and spin rotation

An additional aspect to bear in mind for the dynamic characterization of the magnetization in amorphous ribbons and wires, is the particular magnetic domain formation features of these types of alloys. Such magnetic domain distribution strongly depends on the magnetoelastic coupling between the residual stresses frozen during the rapid solidification process and the sign and magnitude of the alloy magnetostriction λ_s_ [13,14]. For the case of ribbons, longitudinal internal stresses (*i.e*., along the length direction of the sample) develop as a consequence of the gradient of temperature between the wheel contact-side of the ribbon and it’s free-side [3] leading to in-plane-easy axis and hence, longitudinal magnetic domains are preferentially formed for Fe-based ribbons with high λ_s_ constants (λ_s_ ~ 10–30 ppm). In contrast, negative sign of λ_s_ favors the appearance of transverse magnetic domains (*i.e*., perpendicularly oriented respect to the ribbon main axis). Nevertheless, for both cases, the residual stresses follow a rather complex distribution, including in-plane tensile and compressive stresses, but also planar compressive stress inducing perpendicular easy axis (relative to the ribbon surface). These internal stresses can vary not only laterally, but also in depth, leading to the formation of rather complicated magnetic domain configurations including: Wide curved domains with 180° walls, narrow fingerprints, maze and dense stripe domains [15,16]. On the other hand, for amorphous wires the magnetoelastic properties are also determined by the complex quenched-in stresses configuration arising from the casting process, especially from the strong thermal gradient between the inner and the outer regions of the wire, together with the sign of λ_s_. Additionally, for amorphous glass covered microwires, there are two kinds of stress sources (a) axial stress due to the cooling process and (b) radial stress caused by the difference in thermal expansion coefficients between the metallic nucleus and the glass coating [17,18]. These internal stress variations follow a rather complex distribution with axial, circular and radial components changing rapidly from positive (at the axial zone) to negative (at the surrounding shell) including maximum values at half the radius, as well as on the wire edges, and even zero stress value is expected at the precise microwire centre [19,20]. In any case, the sign of λ_s_ determines again the peculiar magnetic domain configuration for these kinds of amorphous alloys: An inner zone with magnetic axial orientation surrounded by a sheath of perpendicular magnetization with either radial (a characteristic of Fe-based wires with large positive λ_s_) or circular orientation (typical of Co- and CoFe-based wires with negative λ_s_) [18,21]. In this work, recent progress on the magnetization dynamics, in terms of the resolution of magnetization processes as a function of frequency, for amorphous ribbons and wires, is presented and discussed

## 2. Results and Discussion

### 2.1. Amorphous Ribbons

**Figure 1:**
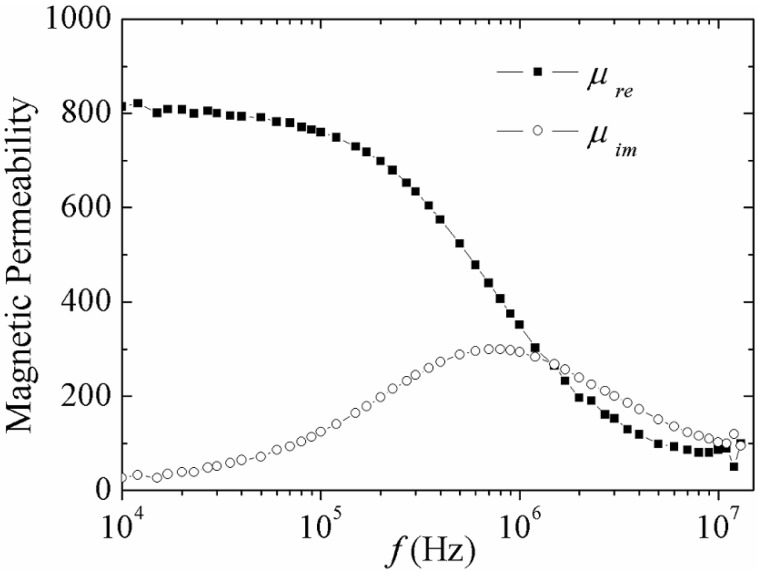
Spectroscopic plots of the complex permeability components for amorphous Fe_80_B_10_Si_10_ ribbon at *h*_ac_ = 0.42.

The spectroscopy behavior for real and imaginary components of complex permeability for amorphous Fe_80_B_10_Si_10_ ribbons are shown in Figure 1. These curves afford the resolution of the active magnetization mechanisms across the frequency range for a given *h*_ac_ intensity [22,23]. For instance, the *μ*_re_(*f* ) plot for an *h*_ac_ = 0.42 A/m shows an initial plateau-like behavior for increasing frequency *f* values up to 6 x10^4^ Hz. This tendency of *μ*_re_ can be attributed to the reversible bulging of the magnetic domain walls (DWs) pinned at the ribbon surface´s defects (such as voids, surface irregularities and the very surface itself). Hence, *μ*_re_ at this part of the frequency is then associated with the initial permeability *μ*_ini_ of the material [22,24] Further, increase in *f* causes a significant reduction in *μ*_re_(*f* ), which can be ascribed to a relaxation-type dispersion of the reversible bulging mechanism for which the DWs are no longer able to follow the ac magnetic variations. Beyond a threshold frequency *f*_x_ (or relaxation frequency) *μ*_re_ becomes very small, reflecting the contribution of the spin rotation as the only magnetization process active for *f* > *f*_x_ [22,24]. Complementarily, the imaginary *μ*_im_ component is attributed to the alloy magnetic losses (hysteresis, eddy current, power losses) [25,26]. The maximum in *μ*_im_ corresponds to *f*_x_.

**Figure 2:**
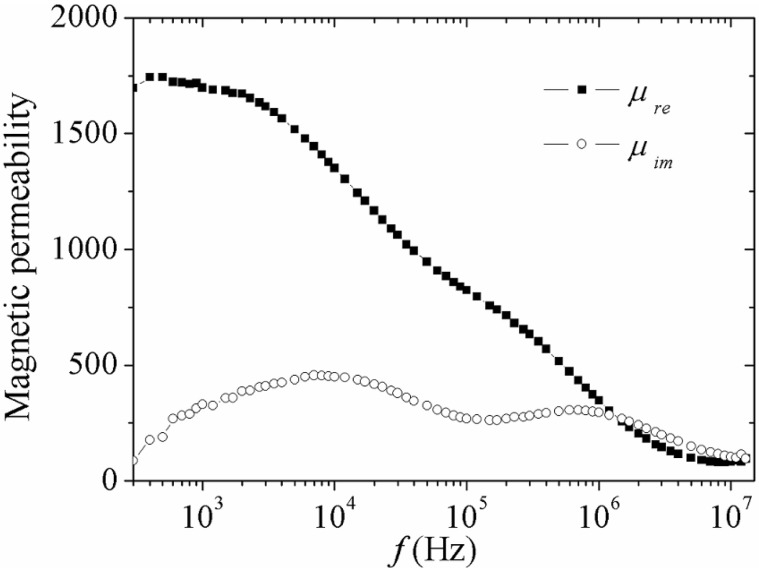
Spectroscopic plots of the complex permeability components for amorphous Fe_80_B_10_Si_10_ ribbon at *h*_ac_ = 1.69 A/m.

On the other hand, for a higher *h*_ac_ intensity of 1.69 A/m, it is possible to observe two sections of the *μ*_re_ (*f*) plot as is shown in Figure 2 for the same FeBSi ribbons: The reversible bulging process, together with its corresponding relaxation for *f* > 1 × 10^5^ Hz, and a significant increase in *μ*_re_ for the frequency interval 3 × 10^3^ Hz < *f* < 1 × 10^5^ Hz, with a precedent constant tendency for *f* < 3 × 10^3^ Hz. This increment in *μ*_re_ can be interpreted in terms of a magnetization mechanism involving the irreversible displacement of DWs caused by the higher *h*_ac_ intensity [22,23,24]. The DWs displacement leads to the hysteresis process within the material, which also presents a relaxation-type dispersion (*i.e*., a change of process) for the aforementioned frequency interval (3 × 10^3^ Hz < *f* < 1 × 10^5^ Hz), for which a progressive decrease in *μ*_re_ is manifested for increasing *f* up to a threshold frequency known as “hysteresis relaxation frequency” *f*_x_^h^. Beyond this *f*_x_^h^, the irreversible displacement of DWs becomes unable to follow the exciting *h*_ac_ field, allowing the reversible bulging to be the active magnetization process because of its shorter time constant. Complementarily, the imaginary component *μ*_im_ (*f* ) exhibits a behavior consistent with the sequence of magnetization mechanisms described already, showing one maximum for each process, *i.e*., irreversible displacement of DWs at *f*_x_^h^ and reversible bulging of DWs at *f*_x_, respectively. For this case *f*_x_^h^ = 8 × 10^3^ Hz and *f*_x_ = 8 × 10^5^ Hz. The marked order of magnitude between both threshold frequencies (*f*_x_^h^ << *f*_x_) reflects the significant difference between the time constant for each magnetization mechanism (much larger for the hysteresis process compared with the reversible bulging of DWs). In addition, the magnetic losses (proportional to the area under the *μ*_im_ curve) are also comparatively larger for the irreversible displacement of DWs than for the reversible bulging, due to the higher energy cost for the hysteresis process relative to the reversible deformation of the DWs.

**Figure 3:**
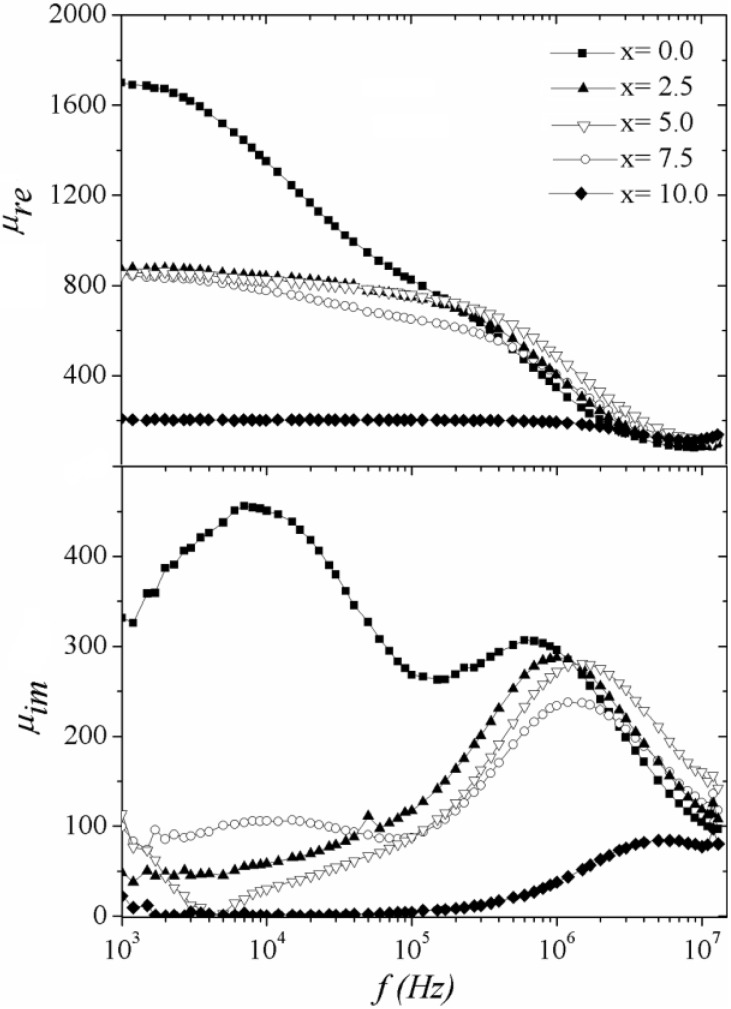
Spectroscopic plots of the complex permeability components for the amorphous ribbon Fe_80_B_10_Si_10-x_Ge_x_ series at *h*_ac_ = 1.69 A/m.

The magnetic permeability and thus, the magnetization processes, are highly sensitive to the chemical composition of the alloy ribbons because of the possible effect on the short-range atomic ordering introduced by variable atom species, which may affect the exchange interaction between magnetic moments as well as the formation of spin-up/spin down bands [27,28,29]. In Figure 3, the real permeability spectroscopic plots (Figure 3a) for melt spun Fe_80_B_10_Si_10-x_Ge_x_ (x = 0.0–10.0) ribbons shows an initial behavior (x = 0.0) including irreversible DWs displacement for *f* < 1 × 10^5^ Hz and its further reversible bulging response for higher frequencies. Nevertheless, for increasing Ge concentration (x ≥ 2.5) the low time constant processes (*i.e*., hysteresis) disappears, leaving the reversible bulging of DWs as the only active mechanism, for which a progressive deleterious tendency is observed with increasing Ge content. Complementarily, the imaginary component *μ*_im_(*f*) (Figure 3b) also exhibits a transition from a double-peak behavior corresponding to two magnetization processes, to a single maximum regime associated to the magnetization mechanism with higher time constant. Additionally, it is possible to observe that the relaxation frequency *f*_x_ increases in a monotonous manner with increasing x, as indicated by the maxima in *μ*_im_(*f*) plots moving to higher frequency values. These results point to a significant compositional influence on the time constant response of amorphous alloys via the effective magnetic anisotropy, which has been observed also in amorphous wires [30] and soft ferrites [31].

**Figure 4:**
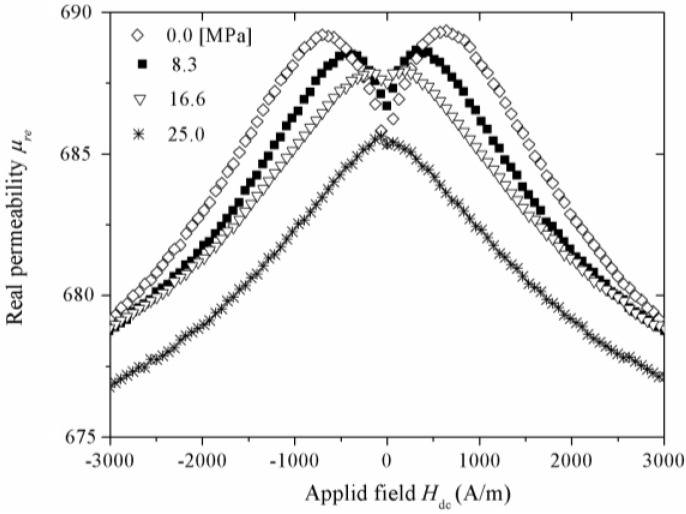
*μ*_re_(H_dc_) curves under the effect of variable tension stress σ for the Fe_52_Co_10_Nb_8_B_30_ ribbon.

As was mentioned in the “Introduction” section, the magnetoelastic coupling between the residual stresses and the alloy magnetostriction λ_s_ plays a major role in determining the magnetic domain configuration of ribbons and wires and, hence, of their magnetic properties. In order to monitor the magnetoelastic coupling for the ribbon series Fe_52−x_Co_10+x_Nb_8_B_30_ (x = 0, 12, 24, 36), *μ*_re_ plots as a function of an applied magnetic dc field H_dc_ and under the effect of variable longitudinal applied tension stress σ at a constant frequency *f* = 13 MHz were determined for each composition. Figure 4 illustrates *μ*_re_ (H_dc_)-σ curves for the Fe_52_Co_10_Nb_8_B_30_ alloy (x = 0), for which at σ = 0, *μ*_re_ initially increases for H_dc_ values between 0 and 800 A/m, followed by a monotonous decrease for H_dc_ > 800 A/m. This behavior is symmetrical for negative H_dc_. For increasing σ, this original two-peak behavior progressively evolves to a reduced single-maximum *μ*_re_ (H_dc_) picture. Same tendencies were observed for the remaining alloy samples, except for the x = 36 alloy, which showed negligible magnetic permeability values as a consequence of its vanishing *λ*_s_ constant.

**Figure 5:**
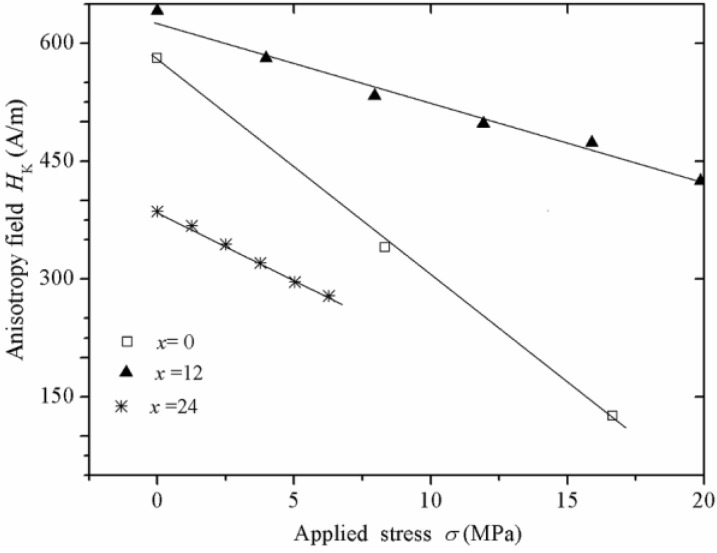
Anisotropy field *H*_k_ as a function of applied stress σ for the Fe_52−x_Co_10+x_Nb_8_B_30_ alloy series. The straight lines correspond to linear fittings with at least R^2^ = 0.99.

The physical meaning of these *μ*_re_ (H_dc_) -σ plots can be explained as follows: At the frequency of measurement (*f* = 13 MHz >> *f*_x_), the spin rotation remains as the only active magnetization mechanism and thus, the real component of permeability corresponds to the rotational permeability *μ*_re_^rot^. For the stress-free curve, the initial increase of *μ*_re_^rot^ up to H_dc_ = 800 A/m can be ascribed to an additional component (axially oriented) of *μ*_re_^rot^ exerted by H_dc_. The maximum in *μ*_re_^rot^ is a consequence of a counterbalancing effect in the rotational magnetization process between the axial H_dc_ and the anisotropy field *H*_k_ in the transverse direction induced during the ribbon fabrication. Thus, the maximum in *μ*_re_^rot^ (H_dc_) corresponds to H_k_ [32,33]. For H_dc_ beyond 800A/m, the rotational permeability decreases since the magnetic spins are mostly axially oriented after having overcome the original anisotropy. On the other hand, for the *μ*_re_^rot^ (H_dc_) curves with increasing σ (up to 16.6 MPa), a shifting of *H*_k_ towards lower values is observed as a consequence of a progressively higher induced axial anisotropy, allowed by the increment of σ, which counterbalance the original transverse anisotropy and thus, the as-cast transverse magnetoelastic coupling. For σ = 25.0 MPa, the alloy transverse anisotropy is averaged out, which results in a single-peak regime with reduced *μ*_re_^rot^ as a consequence of the predominant axial anisotropy induced by σ. This behavior is indicative of a positive *λ*_s_. The dependence of *H*_k_ on σ for alloy ribbons with x ≤ 36 is displayed in Figure 5, for which the following linear trends are manifested (after appropriate linear fittings): H_k_ = 576 [A/m] –27.3 × 10^−6^ [A/(mPa)] σ x = 0; H_k_ = 628 [A/m] –10.3 × 10^−6^ [A/(mPa)] σ, x = 12; and H_k_ = 387 [A/m] –17.7 × 10^−6^ [A/(mPa)] σ, x = 24.

From the functional relations stated above, it is possible to estimate the *λ*_s_ constant for each alloy according to the following relationship [33,34]:

(3) $$\lambda_{s}=-\left(\frac{\mu_{o}M_{s}}{3}\right)\left(\frac{\Delta H_{z}}{\Delta \sigma }\right)$$

where μ_0_*M*_s_ is the saturation magnetization and ΔH_z_ corresponds to the variations of the transverse field H_z_ necessary to compensate the magnetoelastic contribution induced by σ. This equation is proposed for positive and negative *λ*_s_ values [33,34]. For present measurements, the compensating ΔH_z_ field corresponds to *H_k_* straightforward. Therefore, Equation (3) becomes:

(4) $$\lambda_{s}=-\;\left(\frac{\mu_{o}M_{s}}{3}\right)\left(\frac{H_{k}}{\Delta \sigma }\right)$$

Taking μ_o_ = 12.56 × 10^−7^ H/m and the following μ_0_*M*_s_ values determined from *M-H* measurements in a VSM system: 1.24 T (x = 0), 1.09 T (x = 12), 0.86 T (x = 24), in addition to *H*_k_/Δσ as the slopes of the H_k_(σ) plots (Figure 5), we found a decreasing *λ*_s_ tendency with increasing Co concentration as follows: λ_s_ = 10.7 ppm (x = 0), λ_s_ = 3.6 ppm (x = 12) and λ_s_ = 3.0 ppm (x = 24).

### 2.2. Amorphous Wires

**Figure 6:**
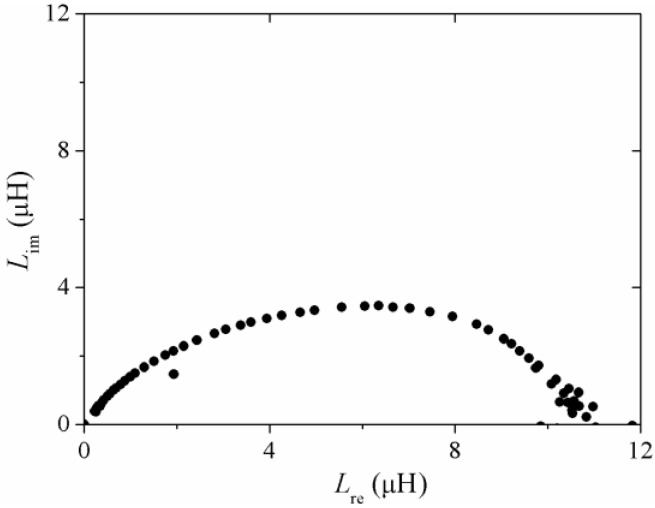
Cole-Cole representation for the complex inductance formalism of (Co_0.94_Fe_0.6_)_72.5_B_15_Si_12.5_ amorphous wire.

An alternative representation of the spectroscopic μ_re_ (*f*), μ_im_ (*f*) plots (or alternatively *L*_re_ (*f*), *L*_im_ (*f*) plots) is the so-called Cole-Cole diagram in which both components are plotted simultaneously in the complex plane, with the frequency *f* being now an implicit variable (the corresponding *f* for each data point increases from right to left in the plot) as is shown in Figure 6 for an amorphous wire of composition (Co_94_Fe_6_)_72.5_B_15_Si_12.5_ (120 μm diameter). For this representation, the formation of well defined semicircles is associated with a magnetization process [23]. In the case of Figure 6, a single semicircle, somehow distorted, reveals the reversible bulging of DWs within the wire as the active mechanism for the frequency range of measurements (10 Hz–13 MHz). The dispersion of the process for the Cole-Cole representation occurs at the middle point onto the semicircle. For this case, *f*_x_ = 88.5 kHz. The distortion of the semicircle reflects a distribution of time constants within the wire, *i.e*., variations of the DWs pinning distance. For a homogenous DWs pinning distance, a perfect circular curvature is expected to occur in the Cole-Cole plane [23].

A more physical insight into the magnetization processes involved in InS measurements can be attained by means of direct calculation of the circular magnetization *M*_φ_ of amorphous wires, which is afforded by the circular magnetic domains characteristic of these alloys. The M_φ_ determination is described as follows:

The magnetic permeability calculation of Equation (2) corresponds to circular permeability *μ*_φ_ in amorphous wires, since the applied magnetic field *h*_ac_ is also of circumferential character as described in the “Experimental Techniques” section. Once *μ_φ_* is known, the wire´s circular induction B_φ_ can be worked out from the permeability definition:

*μ_φ_* = B_φ_/*h_ac_* (5)

and from the general equation

B_φ_ = μ_o_ (M_φ_ + *h_ac_*) (6)

with μ_o_ the vacuum’s permeability.

The calculation of the circular magnetization μ_o_M_φ_ is straightforward according to:

μ_o_M_φ_ = *h_ac_* (*k*L_r_ − μ_o_) (7)

**Figure 7:**
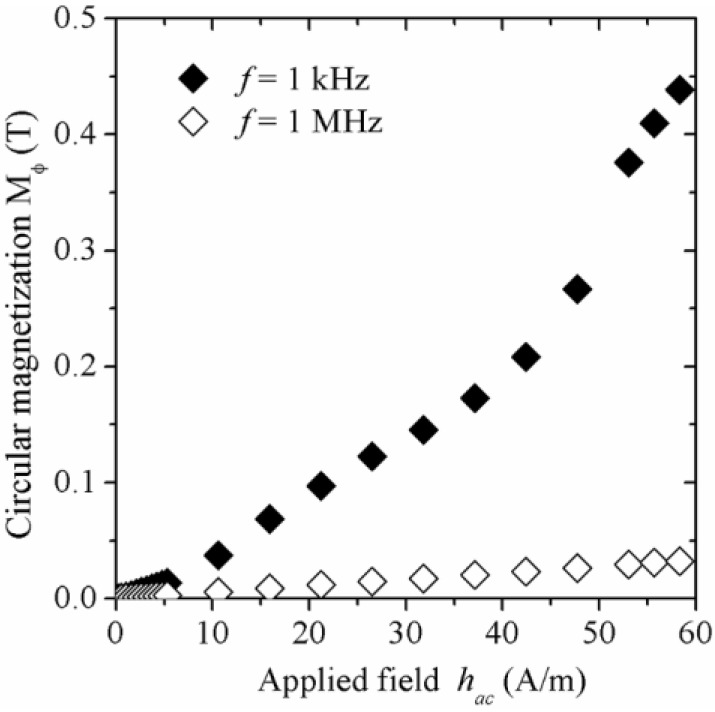
Circular magnetization plots M_φ_ (*h_ac_*) for (Co_0.94_Fe_0.6_)_72.5_B_15_Si_12.5_ amorphous wire.

Therefore, circular magnetization plots μ_o_M_φ_ (*h_ac_*) can be calculated for various *h_ac_* amplitudes at fixed frequencies *f* of *h_ac_*, as is shown in Figure 7, for an amorphous (Co_94_Fe_6_)_72.5_B_15_Si_12.5_ wire with vanishing λ_s_. At low frequency (*i.e*., *f* << *f_x_* ), a typical domain wall magnetization process is observed at the linear initial M_φ_-*h*_ac_ behavior, corresponding to reversible pinned domain wall bulging, followed by a sudden increase in μ_o_M_φ_ at a particular *h_ac_* field value known as the propagation field *h_p_* for which the irreversible wall displacement begins. Further increase in *h_ac_* amplitude should exhibit a μ_o_M_φ_ trend to saturation, where the spin rotation will complete the magnetic moment alignment towards *h_ac_* orientation. On the other hand, for *f* >> *f_x_*, the linear character of the magnetization curve across the *h_ac_* amplitude variation, clearly reflects the spin rotation as the only magnetization mechanism remaining active at such a frequency.

**Figure 8:**
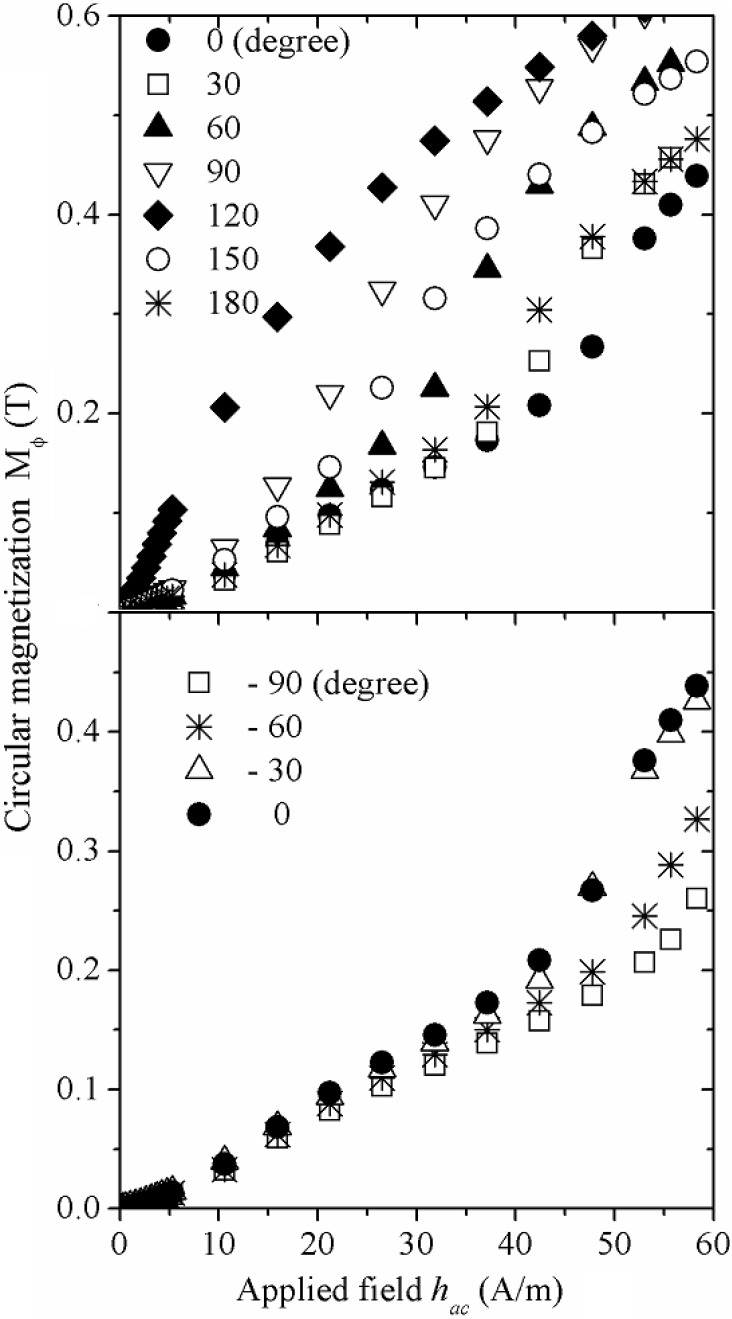
M_φ_ (*h_ac_*) plots for (Co_0.94_Fe_0.6_)_72.5_B_15_Si_12.5_ amorphous wire at variable positive and negative torsion angles.

An interesting set of measurements aiming to determine the effect of torsion strain ζ on the wire´s circular magnetization at low frequency (*f* = 1 kHz) is shown in Figure 8. For positive ζ (clockwise rotation, Figure 8a), μ_o_M_φ_ *versus* *h_ac_*, increases with the torsion angle up to a maximum of 120°. Further increase in ζ results in decreasing μ_o_M_φ_. Counterclockwise rotations (−ζ, Figure 8b), on the other hand, resulted in decreasing μ_o_M_φ_ as ζ increases.

**Figure 9:**
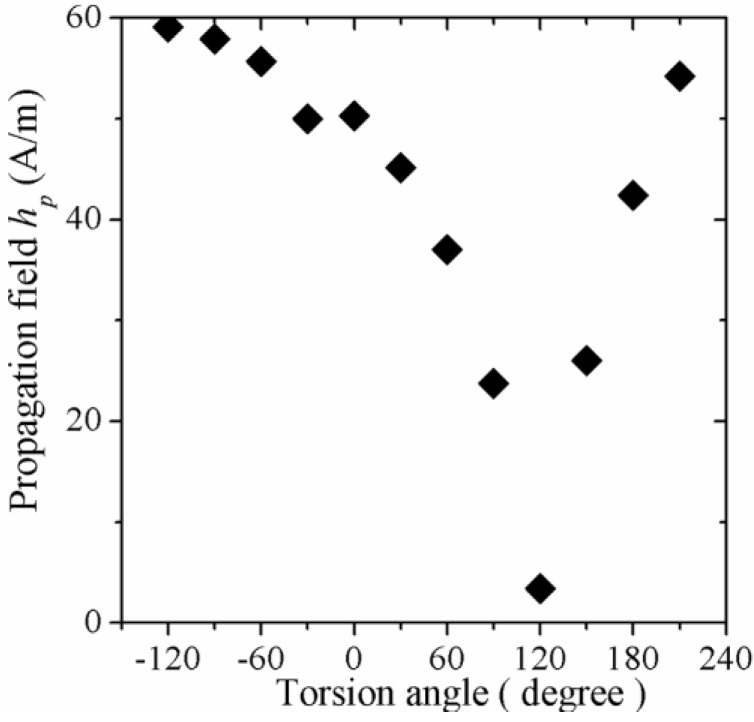
Propagation field *h_p_* as a function of the torsion angle for amorphous (Co_0.94_Fe_0.6_)_72.5_B_15_Si_12.5_ wire.

In addition, *h_p_* (calculated at the inflexion point of each μ_o_M_φ_(*h_ac_*) plot), exhibits a sharp minimum (Figure 9) at the same torsion angle. This particular μ_o_M_φ_, *h_p_* behavior with varying torsion angles can be interpreted in terms of an intrinsic helical anisotropy induced during the fabrication process, which results in internal stresses τ, of about 60 MPa, according to theoretical predictions based on cylindrical layer solidification models [35,36]. Then, as +ζ increases, a counterbalance effect of this +ζ against the internal stress is evident, since the wire becomes magnetically softer, which affords higher μ_o_M_φ_ values (due to higher *μ_φ_*) together with lower *h_p_* values, as a consequence of an enhancement of the unpinning-displacement domain wall process. In contrast, for negative torsion angles, progressive increase in induced anisotropy due to −ζ results in decreasing *μ_φ_* and, hence, in lower μ_o_M_φ_ values, is in agreement with recent reports [37]. The corresponding applied torsion stress τ_appl_, for a torsion angle of 120°, can be calculated as follows:

τ = Kζr
(8)

where K is the wire’s shear modulus, ζ = (torsion angle)/(wire length) is the torsion strain and r is the wire radius. Using K = 60 GPa [38], we have τ_appl_ = 83 MPa, which is in good agreement with the expected internal τ induced during the wire fabrication (between 50 and 100 MPa [39]). Similar τ_appl_ for enhanced *μ_φ_* at various ζ values has also been found in Fe-based wires [40].

### 2.3. Amorphous Glass Coated Microwires

**Figure 10:**
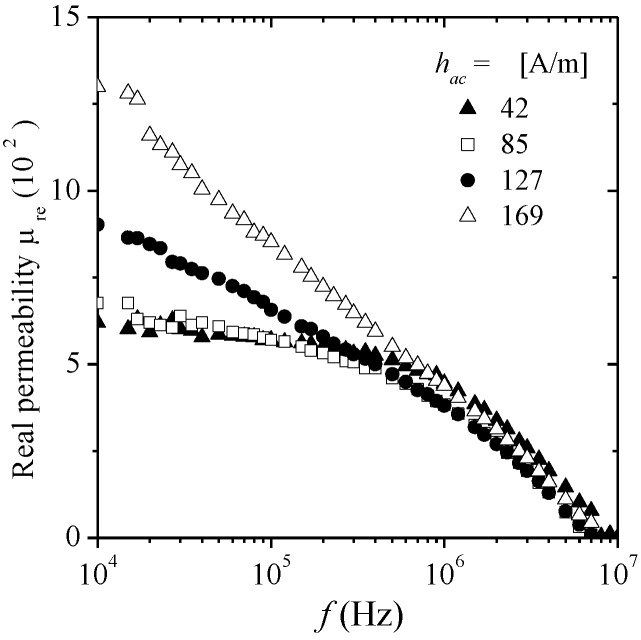
Spectroscopic plots *μ*_re_ (*f*) for glass coated Co_69.7_Fe_3.8_B_13_Si_11_Mo_1.5_Ni_1_ microwire with *h*_ac_ amplitude as a parameter.

**Figure 11:**
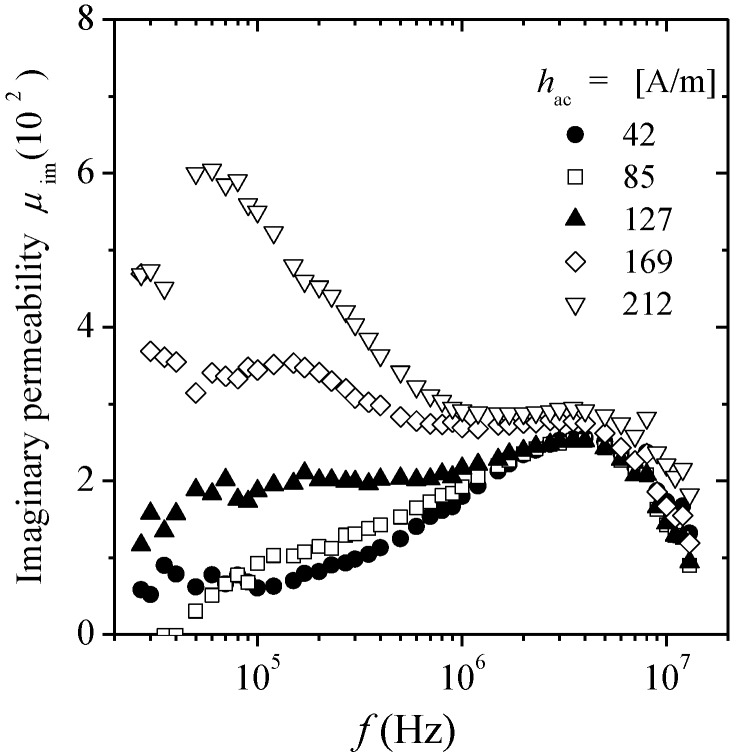
Spectroscopic plots μ_im_ (*f*) for glass coated Co_69.7_Fe_3.8_B_13_Si_11_Mo_1.5_Ni_1_ microwire at various *h*_ac_ field values.

Spectroscopic plots of *μ*_re_ (*f* ) for amorphous Co_69.7_Fe_3.8_B_13_Si_11_Mo_1.5_Ni_1_ glass coated microwires (24 μm diameter, 10μm glass coating) at selected amplitudes of *h*_ac_, are shown in Figure 10. At low fields (*h_ac_* < 127 A/m) and low frequencies (<1 MHz), *μ*_re_ exhibits the same constant trend even for different *h_ac_* amplitudes, followed by its corresponding dispersion. The relaxation frequency *f*_x_ again represents the frequency for which the initial magnetization mechanism is no longer active. This *f_x_* can be determined at the corresponding *μ*_im_ maximum (Figure 11). For the present case, *f_x_* = 2.5 MHz. This *f_x_* value is much higher than the typical *f*_x_ values of amorphous wires with larger diameters values (of about 100–140 μm) with characteristic *f*_x_ ~ 90 kHz. The substantial increase of *f*_x_ for amorphous glass coated microwires is a consequence of their reduced diameter, since the DWs lengths are smaller than those in larger amorphous wires, and therefore, the expected relaxation occurs at higher frequencies. For *h*_ac_ > 127 A/m, *μ*_re_ is no longer independent of *h*_ac_ amplitude (at *f* < *f*_x_). For *f* > *f*_x_, all the plots converge into the low field plot exhibiting the same relaxation dispersion. Assuming a domain structure consisting of circumferential walls for these Co-based microwires, the magnetization process for *h*_ac_ < 127 A/m is associated with reversible bulging of domain walls since these DWs are considered to be pinned to surface defects.

The frequency response of *μ*_im_ for various *h*_ac_ amplitudes is shown in Figure 11. For *h*_ac_ < 127 A/m, a single peak is observed, which is ascribed to the energy dissipated during the reversible domain wall bulging process, whereas for increasing *h*_ac_ such peak remains, but the additional low-frequency peak corresponding to the hysteretic mechanism appears. The height of hysteretic peak increases as *h*_ac_ increases, because the higher the field, the easier DWs displacement, for which larger energy dissipation occurs (compared with domain bulging) leading to the observed higher peaks for increasing *h*_ac_ fields.

**Figure 12:**
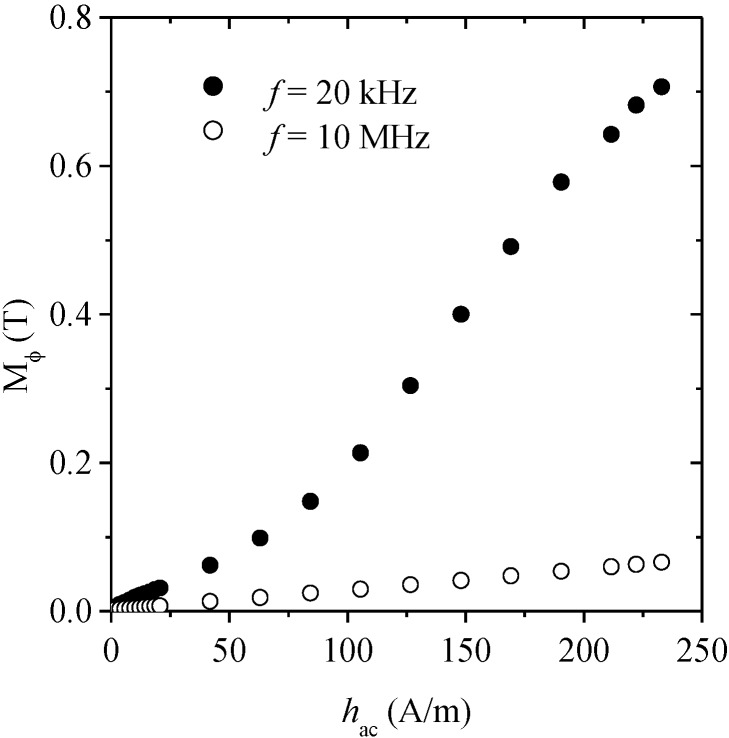
Circular magnetization curves for glass coated Co_69.7_Fe_3.8_B_13_Si_11_Mo_1.5_Ni_1_ microwire at low (*f* << *f*_x_) and high (*f* >> *f*_x_) frequency values.

Circular magnetization curves M_φ_ (*h_ac_*) at two selected frequencies are shown in Figure 12. The low frequency (*f* = 20 kHz) curve exhibits the expected features of domain wall magnetization, such as a rather low initial susceptibility (for *h_ac_* below 100 A/m) followed by a marked increase in slope at about *h_a_*_c_ = 100 A/m, together with a trend to saturation above 200 A/m. In contrast, the curve obtained at 10 MHz (high frequency) shows a linear tendency in agreement with a pure rotational magnetization process.

**Figure 13:**
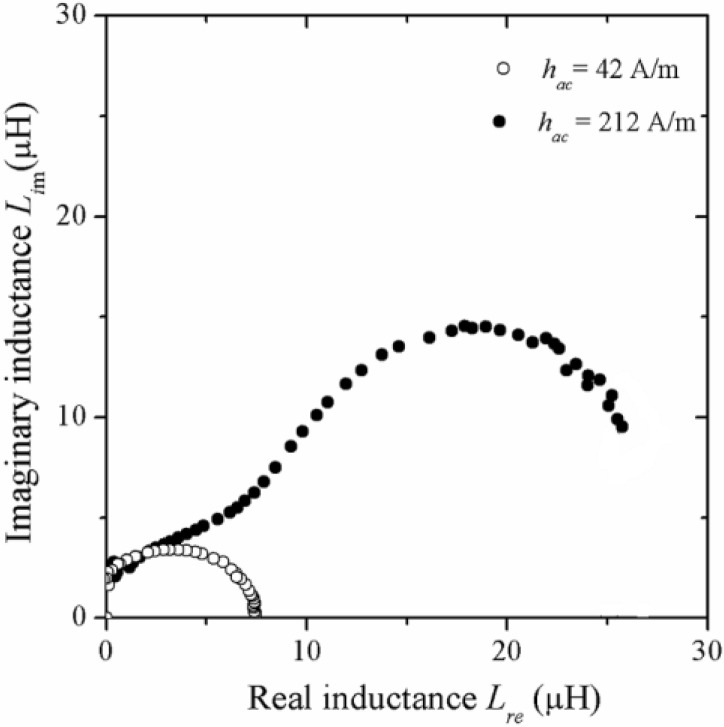
Cole-Cole plots at different *h_ac_* amplitudes for glass coated Co_69.7_Fe_3.8_B_13_Si_11_Mo_1.5_Ni_1_ microwire.

The Cole-Cole representation for the reversible bulging of DWs and the hysteresis process for the same glass coated Co_69.7_Fe_3.8_B_13_Si_11_Mo_1.5_Ni_1_ microwire is depicted in Figure 13 for two distinct h_ac_ amplitudes of 42 A/m and 212 A/m. A well defined semicircle formation (each point obtained at a different frequency) is observed for *h*_ac_ = 42 A/m with a diameter of 7.5 μH associated to the former magnetization mechanism, whilst for *h*_ac_ = 212 A/m, two half-circles of different diameter are evident, the larger one being of 15 μH diameter and for which the irreversible displacement of DWs is attributed.

### 2.4. Biphase Wires

**Figure 14:**
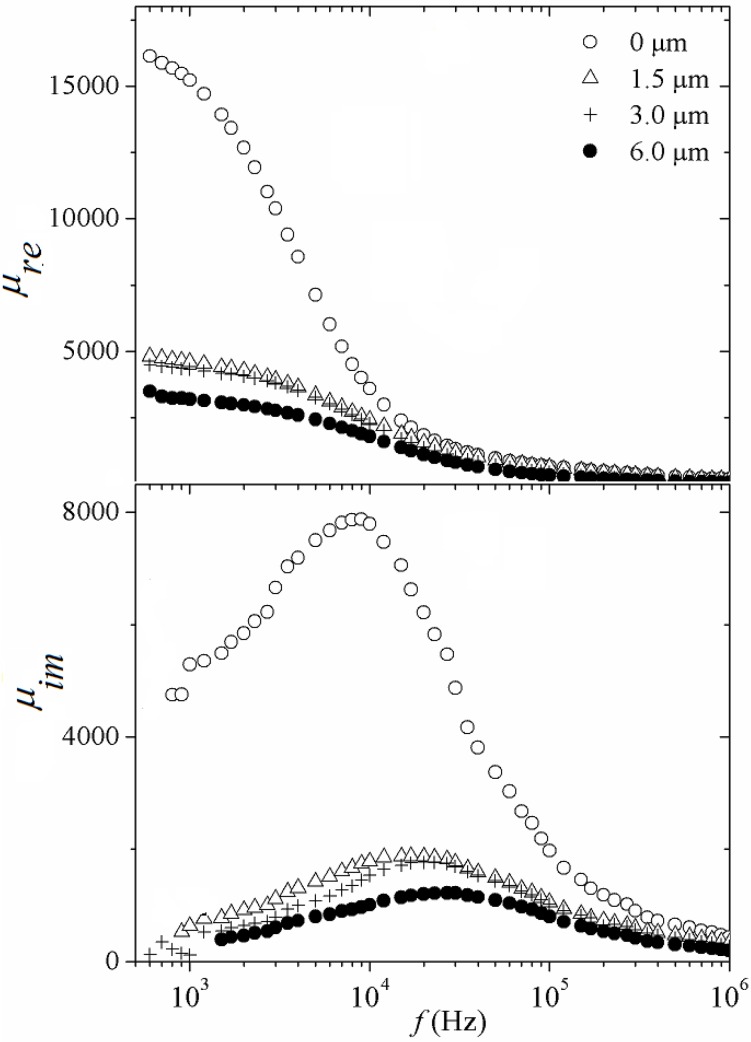
Real and imaginary permeability spectra for biphase wires with hard layer thickness *t* (microns) as a parameter.

Recently, amorphous glass coated microwires have elicited considerable interest for the development of biphase microwires consisting of an amorphous soft magnetic metallic nucleus (obtained by Taylor-Ulitovsky technique) surrounded by a hard magnetic layer deposited by electrochemical process on the glass layer [41]. This hard magnetic layer affords the control of the biphase microwires magnetic properties by means of its magnetic interaction with the soft magnetic nucleus through: (i) the modification of the amorphous wire easy axis of magnetization from radial to longitudinal orientation depending on the hard layer thickness, (*i.e*., on the electrochemical deposition parameters, namely current density and electrochemical exposure time); (ii) the possibility for obtaining controllable magnetostatic or eventually exchange bias response (similar to the bias effect used in spin valves); and (iii) the tailoring of their magnetic domain formation and thus, their magnetic performance from non-hysteretic to bistable behavior [42]. However, biphase systems entailing an amorphous wire nucleus (obtained by rotating water bath melt spinning method) have not yet been explored at all. The spectroscopy response for real and imaginary components of complex permeability for biphase wires composed of an amorphous wire nucleus of composition Fe_64_Co_8_B_19.2_Si_4.8_Ta_4_ (140 μm diameter) and electrodeposited Co_90_Ni_10_ magnetic hard layers with variable thickness *t* of 1.5 μm, 3 μm and 6 μm (attained by carefully control of exposure time to the electrodeposition process) is shown in Figure 14. For the *μ*_re_(*f* ) plot of *t* = 0 μm (Figure 14a), the reversible bulging of the magnetic DWs is manifested for increasing frequency *f* values up to 10^3^ Hz. This part of the real permeability curve corresponds to the initial permeability *μ*_ini_ of the soft magnetic amorphous wire core. Further increase in *f* leads to relaxation of the reversible bulging mechanism at *f* = 9 kHz , leaving the spin rotation as the only magnetization process active for *f* > *f*_x_. The hard layer thickness significantly affects the low-field reversible magnetization process of the soft core since *μ*_ini_ decreases from 16,738 (*t* = 0) to 3,205 (*t* = 6 μm). This marked reduction for *μ*_ini_ can be attributed to the increasing out-of-axis anisotropy, developed by the biphase wires with increasing hard layer thickness, as a consequence of the accumulation of mechanical stress appearing at the amorphous-wire/CoNi-layer interface, for which the thicker the layer, the higher the internal stress accumulated.

Alternatively , the imaginary *μ*_im_ component (Figure 14b) is associated with the magnetic losses (hysteresis, eddy current or power losses) [25]. Consequently, the presence of the external hard layer significantly reduces the losses. The maximum in *μ*_im_ is observed at frequency *f*_x_ for each biphase wire. The *f*_x_ exhibits an increasing tendency (Figure 14b) from 9 kHz (*t* = 0 *μ*m) to 27 kHz (*t* = 6 *μ*m) and follows an inverse tendency with *t*, which confirms the influence of the variable anisotropy on the spin rotation mechanism active for *f* > *f*_x_, for which the product *μ*_re_*f*_x_ = constant is known as the Snoek’s limit [26].

## 3. Experimental Section

Melt spun ribbons with compositions Fe_80_B_10_Si_10−x_Ge_x_ and Fe_52−x_Co_10+x_Nb_8_B_30_ were prepared by means of chill block melt spinning technique with a roll speed of 35 m/s, from initial ingots obtained from arc-melting of pure (99.99%) constituent elements in a Ti-gettered, protective Ar atmosphere. On the other hand, as-cast amorphous (Co_0_._94_Fe_0_._6_)_72.5_B_15_Si_12.5_ wires of 120 μm diameter were prepared by the rotating water bath melt spinning method by using a water depth of 19 mm and an angular drum velocity of 370 rpm. Additionally, amorphous glass coated microwires, 25 μm metallic diameter and 10 μm glass coating of nominal composition Co_69.7_Fe_3.8_B_13_Si_11_Mo_1.5_Ni_1_ were prepared by the Taylor-Ulitovsky technique.

For impedance measurements, an HP 4192 A impedance analyzer controlled by a PC was used. Our measuring system allows the determination of the complex impedance for 94 discrete frequencies from 10 Hz to 13 MHz together with the rms voltage varying between 0.1 and 1.0 V, which leads to longitudinal ac applied magnetic fields *h*_ac_ within the range 0.40–3 A/m on amorphous ribbons by means of a coil with 100 turns; whereas for amorphous wires and microwires, the rms voltage applied directly on the wire ends afforded ac currents *i_ac_* flowing through the sample within the range 2–22 mA (rms) which, in turn, produce circular *h*_ac_ fields within the range 10–240 A/m.

## 4. Conclusions

Inductance spectroscopy has been employed as a powerful tool for characterization of the magnetization dynamics in amorphous ribbons and wires, by means of the resolution of the magnetization mechanism within the materials, in terms of reversible deformation of pinned domain walls, domain wall displacements and spin rotation, for which characteristic parameters, such as the alloy initial permeability and the relaxation frequencies, indicating the dispersion of each process, can be established. In the case of amorphous ribbons, the reversible bulging of DWs and its further displacement was observed as being dependent on the intensity of the ac applied field, together with a clear dependence of both the magnetic permeability and the relaxation frequency with the chemical composition in FeBSiGe alloys. Further, combined experiments with magnetic dc fields and tensile stress, afforded the estimation of the magnetostriction constant in FeCoNbB ribbons. On the other hand, for amorphous wires of 120 microns diameter, the calculation of their circular magnetization curves also reflect the frequency dependency of the magnetization mechanisms identified, as well as the effect of induced anisotropies (by means of torsion stress). In addition, amorphous microwires exhibited similar features for their magnetization mechanism resolution, with distinctive higher relaxation frequencies (compared with amorphous wires) for both reversible bulging of DWs and their displacement, because of the reduced diameters characterizing these alloys obtained by means of the Taylor-Ulitovsky method. Finally, the typical inverse correlation between the alloy´s initial permeability and its frequency relaxation was verified for biphase wires, for which the variable hard coating thickness also influences the magnetic response.
